# Job satisfaction, psychological well-being, and movement facilitation self-efficacy among primary school classroom teachers

**DOI:** 10.3389/fpsyg.2026.1900901

**Published:** 2026-10-16

**Authors:** Arzu Pekğöz Çeviker, Mahmut Karakaya, Fatma Özoğlu, Yener Aksoy, Varol Tutal, Gamze Dağdeviren, Hüseyin Şimşek, Jindong Chang, Zhuoling Lei, Quanrui Tang

**Affiliations:** 1 Department of Primary Education, Faculty of Education, Kırıkkale University, Kırıkkale, Türkiye; 2 Independent Researcher, Educational Administration, Ankara, Türkiye; 3 Department of Physical Education and Sports Education for People with Disabilities, Faculty of Sports Sciences, Inönü University, Malatya, Türkiye; 4 Yaşar Doğu Faculty of Sports Sciences, Ondokuz Mayıs University, Samsun, Türkiye; 5 Faculty of Health Sciences, Malatya Turgut Özal University, Malatya, Türkiye; 6 Department of Physical Education and Sports, Institute of Health Sciences, Fırat University, Elazığ, Türkiye; 7 School of Physical Education, Southwest University, Chongqing, China

**Keywords:** educational administration, games in physical education and sports, movement and skill education, psychological well-being, school-based physical activity, structural equation modeling, teacher job satisfaction, teacher self-efficacy

## Abstract

**Introduction:**

This study examined the direct and indirect relationships among primary school classroom teachers’ job satisfaction, psychological well-being, and movement facilitation self-efficacy in physical education and games lessons using structural equation modeling (SEM).

**Method:**

A quantitative, cross-sectional correlational design was employed. Data were collected from 320 classroom teachers working in Ankara, Türkiye. The Minnesota Job Satisfaction Scale, the short form of the Psychological Well-Being Scale, and the Teacher Self-Efficacy Scale for Providing Physical Activity in the Classroom were administered. The measurement model was validated through confirmatory factor analysis, and convergent and discriminant validity were established. The structural model was tested using maximum likelihood estimation, and mediation was examined via 5,000 bootstrap resamples.

**Results:**

Results indicated that job satisfaction was strongly and positively associated with psychological well-being (*β* = 0.64, *p* < 0.001), and psychological well-being was significantly and positively associated with movement facilitation self-efficacy (*β* = 0.39, *p* < 0.001). Job satisfaction was also significantly and positively associated with movement facilitation self-efficacy (*β* = 0.27, *p* < 0.001). Bootstrap analysis revealed a significant indirect association through psychological well-being (*β*_indirect = 0.25, 95% CI [0.17, 0.34]). The model accounted for 39% of the variance in movement facilitation self-efficacy.

**Conclusion:**

Findings suggest that teachers’ job satisfaction and psychological resources are important correlates of movement facilitation self-efficacy. By situating self-efficacy within the specific context of physical activity promotion, this study integrates occupational well-being theory with school-based physical activity research and highlights implications for teacher support policies and instructional capacity development.

## 1 Introduction

Physical education (PE) and games lessons constitute one of the most important school-based contexts in which children develop movement competence, establish healthy lifestyle habits, and engage in regular moderate-to-vigorous physical activity (MVPA). Beyond their well-documented contributions to physical health, these lessons also promote psychosocial development, emotional well-being, and lifelong motivation for active living (Morales-Sánchez et al., 2021). International organizations have therefore consistently emphasized the strategic role of schools in addressing the global decline in children’s physical activity levels through high-quality physical education programs (UNESCO, 2015; Ha et al., 2019).

Although national curricula provide the general framework for physical education, the quality and intensity of students’ physical activity largely depend on how teachers implement movement-oriented learning experiences in everyday classroom practice. Teachers’ instructional decisions, classroom management strategies, motivational support, and ability to organize physically active learning environments are closely associated with students’ participation in MVPA (Fin et al., 2019). Consequently, increasing attention has shifted from curriculum design toward understanding the teacher-related factors associated with the effectiveness of physical education instruction (Mendez-Giménez et al., 2021; Sánchez-García et al., 2024).

Unlike many other school subjects, physical education requires teachers to simultaneously manage instructional, behavioral, emotional, and physical demands. Effective teaching in PE extends beyond content delivery and involves continuous movement facilitation, embodied demonstration, classroom energy regulation, and real-time behavioral management. These unique pedagogical characteristics require teachers not only to possess instructional competence but also to maintain sufficient psychological and occupational resources to sustain high-quality teaching practices. Özoğlu et al. (2026) emphasized that the distinctive working conditions of physical education teachers make their professional well-being an essential component of instructional effectiveness, while Ilkim (2024) further argued that occupational experiences substantially shape teachers’ professional functioning within physical education settings.

Accordingly, recent research has increasingly recognized that teachers’ instructional effectiveness depends not only on pedagogical knowledge but also on psychosocial characteristics such as occupational well-being, job satisfaction, and self-efficacy (Yan et al., 2022). Systematic reviews further indicate that although research examining teachers’ job satisfaction and psychological well-being has grown considerably, relatively few studies have investigated how these occupational resources are jointly associated with teachers’ instructional behaviors in physical education (Gazali et al., 2024). Classroom teachers, who are primarily responsible for delivering physical education and games lessons in elementary schools, therefore represent a particularly important population for examining the relationships between occupational and psychological resources and movement-oriented teaching practices (Morales-Sánchez et al., 2021).

### 1.1 Teacher well-being and job satisfaction

Teacher well-being and job satisfaction have become central constructs in contemporary educational research because they are associated not only with teachers’ professional sustainability but also with the quality of instructional practices and student learning outcomes (Diener et al., 2018; Skaalvik and Skaalvik, 2017). Job satisfaction reflects teachers’ positive evaluations of their professional experiences, whereas psychological well-being represents broader aspects of optimal psychological functioning, including positive emotions, purpose in life, and effective personal functioning. Together, these constructs constitute important occupational resources that enable teachers to cope with professional demands, sustain motivation, and maintain instructional quality over time (Istiqomah and Tjalla, 2023; Aznar et al., 2024).

Within physical education, the importance of these occupational resources may be even greater because teaching extends beyond cognitive instruction to include continuous movement facilitation, classroom organization, motivational communication, and active participation in physically demanding learning environments. Consequently, physical education teachers frequently encounter unique occupational challenges, including role strain, emotional labor, professional marginalization, and heavy instructional demands, all of which may influence both their psychological functioning and instructional effectiveness (Carraro et al., 2016; Richards et al., 2019; Yan et al., 2022). Recent systematic reviews further demonstrate that teachers’ professional competence, motivation, and job satisfaction are closely associated with the quality of physical education instruction and students’ participation in physical activity (Gazali et al., 2024; Yan et al., 2022).

Empirical evidence consistently indicates a positive association between job satisfaction and psychological well-being across educational settings. Teachers who report greater satisfaction with their work also tend to experience stronger psychological resilience, more positive affect, higher professional commitment, and lower levels of emotional exhaustion (Dayne et al., 2023; Aznar et al., 2024). From the perspective of positive organizational psychology, job satisfaction is associated with psychological resources that enable teachers to cope more effectively with everyday instructional challenges, thereby supporting sustained teaching performance and professional engagement (Diener et al., 2018; Istiqomah and Tjalla, 2023). These relationships may be particularly relevant for classroom teachers responsible for delivering physical education and games lessons, where maintaining students’ active participation requires continuous instructional energy, motivation, and emotional regulation.

Despite this growing body of evidence, much of the existing literature has examined job satisfaction and psychological well-being either as independent predictors or as isolated outcomes. Comparatively fewer studies have investigated how these occupational resources function together within an integrated explanatory framework in physical education settings, particularly among primary education classroom teachers who are responsible for implementing movement-oriented instruction. This limitation suggests that the relationships linking job satisfaction with teachers’ instructional functioning remain insufficiently understood, thereby justifying further investigation of the associations among job satisfaction, psychological well-being, and movement-related teaching outcomes (Gazali et al., 2024; Yan et al., 2022).

### 1.2 Movement facilitation self-efficacy in primary education

Self-efficacy is one of the central constructs of Bandura’s (1997) Social Cognitive Theory and refers to individuals’ beliefs in their capability to organize and execute the actions required to achieve desired outcomes. Rather than reflecting actual competence, self-efficacy represents individuals’ judgments about their ability to successfully perform specific tasks under particular circumstances. These efficacy beliefs influence motivation, persistence, emotional regulation, instructional decision-making, and performance, especially when individuals encounter challenging situations (Bandura, 1997).

Within educational settings, teacher self-efficacy refers to teachers’ beliefs in their capability to plan, organize, and implement instructional practices that facilitate student learning and achievement (Zee and Koomen, 2016). Teachers with stronger self-efficacy beliefs demonstrate greater instructional persistence, adopt more effective classroom management strategies, are more willing to implement innovative teaching practices, and are better able to motivate students during learning activities (Zee and Koomen, 2016). Consequently, teacher self-efficacy has consistently been identified as one of the strongest psychological predictors of instructional quality and student engagement across educational contexts.

In physical education, however, self-efficacy extends beyond general instructional competence. Teaching movement-based lessons requires teachers to continuously organize learning environments, demonstrate motor skills, regulate students’ physical engagement, ensure safety, and sustain participation throughout physically active lessons. Therefore, movement facilitation self-efficacy represents a domain-specific form of teacher efficacy that reflects teachers’ confidence in their ability to successfully promote, organize, and maintain students’ participation in physical activity during physical education and games lessons (Raven and Pels, 2021; Valenzuela A. et al., 2021; Valenzuela R. et al., 2021). This instructional capacity is particularly important in primary education, where classroom teachers are responsible for delivering physical education despite often having limited specialist training in physical education pedagogy.

Research further suggests that teachers’ efficacy beliefs are closely associated with their psychological functioning. Individuals experiencing higher levels of psychological well-being generally report greater confidence in managing professional responsibilities, overcoming instructional challenges, and maintaining effective teaching practices (Mokhtar et al., 2021; Tsang et al., 2021). Positive psychological functioning is associated with greater emotional regulation, resilience, and adaptive coping strategies, which may also be associated with greater confidence in implementing movement-oriented instruction. Conversely, lower levels of psychological well-being may be associated with lower instructional confidence and less willingness to organize physically active learning experiences.

Existing evidence also indicates that occupational experiences contribute substantially to the development of teacher self-efficacy. Teachers who experience greater job satisfaction tend to report stronger professional commitment, higher perceptions of competence, and greater instructional confidence (Pham et al., 2022; Istiqomah and Tjalla, 2023). Within physical education, supportive working conditions and positive professional experiences have likewise been associated with greater confidence in implementing movement-oriented pedagogical practices (Yan et al., 2022). Nevertheless, although these relationships have been documented independently, limited research has examined how job satisfaction and psychological well-being operate together in explaining movement facilitation self-efficacy among primary education classroom teachers. Understanding these relationships is important because occupational and psychological resources may be jointly associated with teachers’ capacity to provide physically active learning environments that support children’s participation in regular physical activity.

### 1.3 Conceptual gap and research rationale

Although job satisfaction, psychological well-being, and teacher self-efficacy have each received considerable attention within educational and organizational psychology, existing research has predominantly examined these constructs through independent or bivariate relationships. Studies have generally focused on how job satisfaction relates to psychological well-being, or how psychological well-being is associated with teacher self-efficacy, without considering the interconnected relationships among these occupational resources and instructional functioning (Kang et al., 2020; Istiqomah and Tjalla, 2023; Aznar et al., 2024).

Within physical education research, this limitation is even more evident. Existing studies have primarily investigated teachers’ motivation, professional competence, burnout, or job satisfaction separately, whereas relatively few have simultaneously examined how job satisfaction and psychological well-being operate together in explaining teachers’ confidence in facilitating students’ physical activity (Gazali et al., 2024; Yan et al., 2022). Moreover, previous investigations have largely relied on correlational approaches that do not adequately account for measurement error or distinguish between direct and indirect relationships among these psychosocial constructs (Kang et al., 2020).

An additional limitation concerns the conceptualization of teacher self-efficacy. Much of the educational literature measures self-efficacy as a general teaching construct applicable across all instructional settings (Zee and Koomen, 2016). However, movement-oriented instruction presents pedagogical demands that differ substantially from those encountered in conventional classroom teaching. Delivering effective physical education requires teachers to motivate continuous participation, organize dynamic learning environments, regulate students’ physical engagement, and facilitate active movement throughout instruction. Consequently, movement facilitation self-efficacy should be conceptualized as a domain-specific instructional capability rather than as a general perception of teaching competence (Raven and Pels, 2021; Valenzuela A. et al., 2021; Valenzuela R. et al., 2021).

Furthermore, limited evidence is available regarding primary education classroom teachers, who constitute the principal providers of physical education and games lessons in many educational systems. While specialist physical education teachers have received increasing scholarly attention, classroom teachers have been comparatively underrepresented in studies examining movement-oriented instructional efficacy. Understanding how job satisfaction and psychological well-being are associated with this group’s confidence in promoting physical activity is therefore both theoretically and practically important, particularly because these teachers play a central role in establishing children’s lifelong physical activity habits (Morales-Sánchez et al., 2021; Gazali et al., 2024).

To address these gaps, the present study proposes an integrated structural model in which job satisfaction is considered an occupational resource, psychological well-being represents a psychological variable through which an indirect association is statistically specified, and movement facilitation self-efficacy represents the instructional outcome. By examining both direct and indirect associations among these constructs using Structural Equation Modeling (SEM), the study simultaneously accounts for latent measurement error and evaluates the indirect association through psychological well-being. SEM provides a rigorous analytical framework for examining complex psychosocial relationships within a unified model (Kline, 2016).

Accordingly, this study seeks to clarify how occupational experiences are associated with movement-oriented instructional confidence among primary education classroom teachers, thereby extending current knowledge beyond isolated associations toward a more integrated understanding of the relationships among these psychosocial constructs.

### 1.4 Contribution of the present study

The present study makes several theoretical, methodological, and practical contributions to the international literature on physical education and teacher psychology.

First, the study extends existing research by integrating job satisfaction, psychological well-being, and movement facilitation self-efficacy within a single structural framework. Although these constructs have frequently been investigated independently in educational research, empirical studies simultaneously examining their direct and indirect relationships remain limited, particularly within physical education settings (Kang et al., 2020; Gazali et al., 2024). By examining these variables together, the present study provides a more comprehensive understanding of the direct and indirect associations linking teachers’ occupational experiences with instructional functioning.

Second, this study conceptualizes self-efficacy from a domain-specific perspective by focusing on movement facilitation self-efficacy rather than general teacher self-efficacy. Whereas most previous studies have assessed teachers’ overall instructional confidence, the present research emphasizes teachers’ perceived capability to organize, promote, and sustain students’ participation in physical activity during physical education and games lessons. This distinction is important because movement-oriented teaching requires unique pedagogical, motivational, and classroom management competencies that extend beyond general instructional practice (Raven and Pels, 2021; Valenzuela A. et al., 2021; Valenzuela R. et al., 2021).

Third, the study contributes to the physical education literature by focusing specifically on primary education classroom teachers, a population that has received comparatively less attention than specialist physical education teachers despite their central responsibility for delivering physical education and games lessons in many elementary schools. By investigating classroom teachers’ occupational and psychological resources, the study broadens current understanding of the factors supporting effective movement-oriented instruction within primary education.

Methodologically, the study contributes by employing Structural Equation Modeling (SEM) to simultaneously examine measurement and structural relationships while accounting for latent measurement error. Unlike conventional correlational or regression-based approaches, SEM allows the simultaneous estimation of direct and indirect associations within a unified analytical framework, thereby enabling the hypothesized relationships among job satisfaction, psychological well-being, and movement facilitation self-efficacy to be examined concurrently (Kline, 2016).

Finally, the findings offer practical implications for educational leadership and school policy. If teachers’ job satisfaction is associated with movement facilitation self-efficacy both directly and indirectly through psychological well-being, initiatives aimed at supporting teachers’ occupational well-being may also contribute to their confidence in promoting physically active learning environments. Consequently, teacher well-being should be considered not only an individual occupational outcome but also a strategic educational resource that supports the successful implementation of school-based physical activity programs (Gazali et al., 2024).

### 1.5 Hypotheses development

The theoretical framework of this study is grounded in Social Cognitive Theory (Bandura, 1997) and contemporary perspectives on occupational well-being, which collectively suggest that teachers’ professional experiences are associated with instructional outcomes through interconnected psychological processes. Drawing upon this framework and the empirical evidence reviewed above, the present study proposes that job satisfaction, psychological well-being, and movement facilitation self-efficacy are linked through both direct and indirect relationships among primary education classroom teachers.

Previous research has consistently demonstrated that teachers who experience greater job satisfaction also report higher levels of psychological well-being, with positive occupational experiences being associated with greater resilience, positive affect, and professional engagement (Dayne et al., 2023; Istiqomah and Tjalla, 2023; Aznar et al., 2024). Accordingly, the first hypothesis is proposed:

*H1*: Job satisfaction is positively associated with psychological well-being among primary education classroom teachers.

Research further indicates that psychologically healthier teachers demonstrate stronger confidence in managing instructional challenges, motivating students, and implementing effective teaching practices (Bandura, 1997; Zee and Koomen, 2016; Mokhtar et al., 2021; Tsang et al., 2021). Within movement-oriented instruction, these psychological resources are expected to be positively associated with teachers’ confidence in promoting students’ participation in physical activity. Therefore, the following hypothesis is formulated:

*H2*: Psychological well-being is positively associated with movement facilitation self-efficacy among primary education classroom teachers.

Occupational experiences may also be associated with instructional confidence independently of psychological well-being. Teachers who are satisfied with their professional roles generally report stronger perceptions of competence, greater instructional commitment, and higher levels of professional efficacy (Pham et al., 2022; Istiqomah and Tjalla, 2023). Within physical education, positive working conditions and professional recognition have likewise been associated with greater confidence in implementing movement-oriented pedagogical practices (Yan et al., 2022). Accordingly, the following direct relationship is proposed:

*H3*: Job satisfaction is positively associated with movement facilitation self-efficacy among primary education classroom teachers.

Finally, previous studies suggest that job satisfaction may be associated with teachers’ instructional functioning through its relationship with their psychological resources (Kang et al., 2020; Dayne et al., 2023). Building upon this evidence, the present study proposes that psychological well-being may statistically account for part of the association between job satisfaction and movement facilitation self-efficacy. Accordingly, the final hypothesis is stated as follows:

*H4*: Job satisfaction is indirectly associated with movement facilitation self-efficacy through psychological well-being among primary education classroom teachers.

## 2 Materials and methods

### 2.1 Research model

This study employed a quantitative, cross-sectional correlational research design to examine the relationships among classroom teachers’ job satisfaction, psychological well-being, and movement facilitation self-efficacy. Quantitative correlational designs are appropriate for examining theoretically specified associations among variables and testing hypothesized structural relationships based on empirical data (Creswell, 2014; Karasar, 2020).

The conceptual framework of the study was developed on the basis of Social Cognitive Theory (Bandura, 1997), which posits that individuals’ beliefs, psychological characteristics, and environmental experiences interact in relation to behavior. Consistent with this perspective, the proposed model specifies that classroom teachers’ job satisfaction is positively associated with psychological well-being, which, in turn, is associated with higher levels of movement facilitation self-efficacy. Accordingly, the model includes both direct associations and an indirect association between job satisfaction and movement facilitation self-efficacy through psychological well-being. Given the cross-sectional nature of the study, psychological well-being is conceptualized as a variable through which this indirect association is statistically specified, rather than as evidence of a causal or temporal mediating mechanism (Hayes, 2018).

To test the proposed theoretical framework, Structural Equation Modeling (SEM) was employed. SEM is particularly appropriate for testing theoretically driven models because it simultaneously evaluates measurement and structural relationships, controls for measurement error, and estimates direct and indirect effects within a single analytical framework (Kline, 2016; Schumacker and Lomax, 2016). Compared with conventional regression-based approaches, SEM provides more robust parameter estimates when latent constructs are measured using multiple observed indicators and allows the hypothesized indirect association to be estimated within the structural model. Model estimation was conducted using the Maximum Likelihood (ML) estimation method, which is recommended when multivariate normality assumptions are reasonably satisfied (Hair et al., 2019).

### 2.2 Population and sample

The target population of this study comprised classroom teachers employed in public primary schools in Ankara, Türkiye, during the 2024-2025 academic year. Participants were recruited using a convenience sampling approach, which is widely used in educational research when access to participants is determined by institutional accessibility and voluntary participation (Creswell, 2014). A total of 336 questionnaires were initially collected. Following data screening, 16 questionnaires were excluded because of incomplete responses or missing data, resulting in a final analytical sample of 320 classroom teachers.

The adequacy of the sample size was evaluated with reference to recommendations for Structural Equation Modeling (SEM). Previous methodological studies suggest that samples of at least 200 participants are generally sufficient for SEM analyses, whereas samples of 300 or more provide more stable parameter estimates, greater statistical power, and improved model stability (Kline, 2016; Schumacker and Lomax, 2016; Hair et al., 2019). Therefore, the final sample of 320 participants was considered adequate for testing the proposed structural model.

Of the participants, 200 (62.5%) were female and 120 (37.5%) were male. Regarding age, 22.5% were between 31 and 40 years, 47.5% were between 41 and 50 years, and 30.0% were 51 years of age or older. Most participants were married (85.0%), whereas 15.0% were single. Concerning educational attainment, 67.5% held a bachelor’s degree and 32.5% had completed a master’s degree. Professional experience was generally high; 62.5% of the participants had 20 years or more of teaching experience, 25.0% had 16–20 years, 10.0% had 11–15 years, and 2.5% had 6–10 years of professional experience. Overall, these characteristics indicate that the sample consisted primarily of experienced classroom teachers actively responsible for delivering physical education and games lessons in primary schools.

Participants were included in the study if they met the following criteria:

1. They were employed as classroom teachers in public primary schools in Ankara.
2. They were actively responsible for teaching physical education and games lessons.
3. They voluntarily agreed to participate in the study after being informed about its purpose.
4. They completed all measurement instruments without missing responses.

Participation was entirely voluntary, and no financial or professional incentives were provided. All questionnaires were anonymized prior to analysis to ensure participant confidentiality.

### 2.3 Data collection process

Prior to data collection, ethical approval for the study was obtained from the relevant Institutional Ethics Committee (Approval No: 16.01.2026-441406). Permission to conduct the research was also obtained from the relevant educational authorities and school administrations. The study was conducted in accordance with the ethical principles of the Declaration of Helsinki and national regulations governing research involving human participants.

Before completing the questionnaires, all participants were informed about the purpose of the study, the voluntary nature of participation, the confidentiality of their responses, and their right to withdraw from the study at any stage without any negative consequences. Written informed consent was obtained from all participants before data collection commenced.

Data were collected between March and May 2026 using both online questionnaires and paper-and-pencil forms. Employing both modes of administration facilitated access to teachers working in different schools and increased participation by accommodating individual preferences and institutional conditions. Participants completed the questionnaires individually during their available time, and the average completion time was approximately 10–15 min.

A total of 336 questionnaires were returned during the data collection period. Following the initial screening process, questionnaires were examined for completeness and response quality. Sixteen questionnaires containing substantial missing data were excluded from further analyses. Consequently, the final dataset consisted of 320 complete questionnaires that satisfied all inclusion criteria.

To ensure confidentiality, no personally identifiable information (e.g., names, identity numbers, or school identifiers) was collected. All questionnaires were coded numerically before analysis, and the dataset was stored in a secure electronic environment accessible only to the research team. The collected data were used exclusively for scientific purposes and reported only in aggregate form.

#### 2.3.1 Measurement tools

##### 2.3.1.1 Minnesota Satisfaction Questionnaire (MSQ)

Teachers’ job satisfaction was assessed using the 20-item short form of the Minnesota Satisfaction Questionnaire (MSQ) originally developed by Weiss et al. (1967) and adapted into Turkish by Baycan (1985). The MSQ is one of the most widely used instruments for assessing employees’ satisfaction with different aspects of their work and has demonstrated strong psychometric properties across occupational groups.

The Turkish version consists of 20 items organized into two dimensions: Intrinsic Satisfaction (12 items) and Extrinsic Satisfaction (8 items). Intrinsic satisfaction reflects satisfaction derived from the nature of the work itself (e.g., achievement, responsibility, and opportunities for personal growth), whereas extrinsic satisfaction refers to satisfaction associated with external working conditions such as supervision, organizational policies, and salary.

A sample item is “I am satisfied with the opportunity to use my abilities in my job.” Participants responded using a 5-point Likert scale ranging from 1 (Strongly disagree) to 5 (Strongly agree). Higher scores indicate higher levels of job satisfaction.

Baycan (1985) reported satisfactory reliability coefficients for the Turkish adaptation of the scale. In the present study, Cronbach’s alpha coefficients were 0.88 for the Intrinsic Satisfaction subscale, 0.79 for the Extrinsic Satisfaction subscale, and 0.90 for the total scale, indicating excellent internal consistency.

##### 2.3.1.2 Psychological Well-Being Scale

Psychological well-being was measured using the Psychological Well-Being Scale developed by Diener et al. (2010) and adapted into Turkish by Telef (2013). The scale evaluates individuals’ positive psychological functioning, including purpose in life, competence, positive relationships, optimism, and personal growth. The Turkish adaptation demonstrated a single-factor structure, satisfactory construct validity, and good internal consistency (Telef, 2013).

The instrument consists of eight items and is scored on a 7-point Likert scale ranging from 1 (Strongly disagree) to 7 (Strongly agree). An example item is “I lead a purposeful and meaningful life.” Higher total scores indicate higher levels of psychological well-being.

In the Turkish adaptation study, the scale demonstrated satisfactory psychometric properties with a Cronbach’s alpha coefficient of 0.80 and evidence supporting construct validity. In the present study, the overall Cronbach’s alpha coefficient was also 0.80, indicating satisfactory internal consistency.

##### 2.3.1.3 Teacher Efficacy for Teaching Physical Activity in the Primary Classroom Scale (TETPPACS)

Teachers’ movement facilitation self-efficacy was assessed using the Teacher Efficacy for Teaching Physical Activity in the Primary Classroom Scale (TETPPACS) originally developed by Centeio et al. (2022) and recently adapted into Turkish by Karakaya (2025). The scale measures classroom teachers’ confidence in their ability to integrate physical activity into everyday classroom practices and overcome barriers that may hinder movement-oriented instruction.

The Turkish version contains 18 items organized into three dimensions:

- Student barriers (6 items).
- Institutional barriers (9 items).
- Educational barriers (3 items).

The response format is a 5-point Likert scale, ranging from 1 (Strongly disagree) to 5 (Strongly agree), with higher scores indicating stronger movement facilitation self-efficacy.

A representative item is “I can provide opportunities for students to be physically active during the school day despite potential barriers.” The Turkish adaptation study confirmed the original three-factor structure through exploratory and confirmatory factor analyses and reported excellent model fit indices (*χ*^2^/d*f* = 1.03, RMSEA = 0.008, CFI = 0.98, TLI = 0.97), together with high internal consistency coefficients.

In the present study, Cronbach’s alpha coefficients were 0.84 for Student Barriers, 0.92 for Institutional Barriers, 0.94 for Educational Barriers, and 0.94 for the overall scale, indicating excellent reliability.

#### 2.3.2 Overall reliability of the measurement instruments

The internal consistency of all measurement instruments was evaluated using Cronbach’s alpha coefficients. According to widely accepted criteria, alpha values of 0.70 or higher indicate acceptable internal consistency, values above 0.80 indicate good reliability, and values exceeding 0.90 indicate excellent reliability (Hair et al., 2019). The reliability coefficients obtained in the present study ranged from 0.79 to 0.94, demonstrating that all instruments exhibited satisfactory to excellent internal consistency for the study sample.

#### 2.3.3 Data screening

Before conducting the statistical analyses, the dataset was screened for completeness, accuracy, and compliance with the assumptions required for Structural Equation Modeling. Initially, all questionnaires were examined for missing responses and response consistency. Sixteen questionnaires containing incomplete responses were excluded from the analyses. The remaining dataset consisted of 320 complete cases.

Subsequently, the data were examined for univariate and multivariate outliers, missing values, normality, multi-collinearity, and common method bias. The results indicated that the data were suitable for subsequent confirmatory factor analysis and structural equation modeling.

#### 2.3.4 Preliminary analyses

Prior to testing the structural model, the dataset was systematically screened to determine its suitability for Structural Equation Modeling (SEM). Preliminary analyses were conducted to evaluate missing data, outliers, distributional assumptions, multi-collinearity, and potential common method bias before hypothesis testing (Kline, 2016; Hair et al., 2019).

Initially, the completeness of the dataset was examined. The proportion of missing data was below 5%, indicating that missing responses were unlikely to bias the analyses. Furthermore, the Missing Completely at Random (MCAR) test suggested that the missing values were randomly distributed; therefore, listwise deletion was considered appropriate.

Univariate and multivariate outliers were subsequently evaluated. Standardized *z* scores and Mahalanobis distance statistics were examined to identify potentially influential observations. No cases exceeded the recommended cutoff values; therefore, no additional observations were removed prior to the analyses.

The assumption of normality was assessed using both univariate and multivariate indicators. Univariate normality was evaluated through skewness and kurtosis coefficients. All observed variables exhibited skewness and kurtosis values within the recommended range of ±1.5, indicating acceptable univariate normality (Hair et al., 2019). Multivariate normality was further evaluated using Mardia’s coefficient, and the results indicated that the assumption of multivariate normality was sufficiently satisfied to permit the use of Maximum Likelihood (ML) estimation (Kline, 2016).

Multicollinearity was examined using the Variance Inflation Factor (VIF). All VIF values were below 5.00, indicating that multicollinearity did not pose a threat to parameter estimation (Hair et al., 2019).

Because all data were obtained through self-report questionnaires administered at a single point in time, the possibility of common method bias was also evaluated. Harman’s single-factor test was performed by loading all observed variables into an exploratory factor analysis. The first unrotated factor accounted for less than 50% of the total variance, suggesting that common method variance was unlikely to constitute a serious concern in the present study (Podsakoff et al., 2003).

Overall, the preliminary analyses indicated that the dataset satisfied the statistical assumptions required for SEM, supporting the subsequent evaluation of the measurement and structural models.

Although the MSQ and TETPPACS comprise theoretically meaningful subdimensions, the present study examined these measures at the overall construct level. This specification was determined by the primary theoretical objective of the study, which focused on the structural relationships among overall job satisfaction, psychological well-being, and movement facilitation self-efficacy rather than on dimension-specific associations. Accordingly, the subdimensions were acknowledged as components of the respective broader constructs, while the structural hypotheses were specified at the overall construct level. The use of the overall constructs was also supported by the strong internal consistency of the total scores in the present sample (*α* = 0.90 for job satisfaction and α = 0.94 for movement facilitation self-efficacy). This approach should not be interpreted as implying that the respective subdimensions are psychometrically equivalent; rather, dimension-specific relationships were beyond the scope of the present study.

Alternative first-order multidimensional, second-order, and bifactor specifications were not formally compared in the present study because the structural hypotheses were formulated at the overall construct level. Accordingly, the construct-level specification was adopted to align the measurement model with the theoretical scope of the proposed structural relationships.

### 2.4 Data analysis

Data were analyzed using a two-step Structural Equation Modeling (SEM) approach recommended by Anderson and Gerbing (1988). This approach separates the evaluation of the measurement model from the assessment of the structural model, thereby providing stronger evidence for construct validity before testing the hypothesized relationships among latent variables.

In the first stage, Confirmatory Factor Analysis (CFA) was conducted to evaluate the measurement model and to determine whether the observed indicators adequately represented their respective latent constructs. CFA was performed using the Maximum Likelihood (ML) estimation method because preliminary analyses indicated that the assumptions of multivariate normality were reasonably satisfied (Kline, 2016; Hair et al., 2019). Standardized factor loadings (*λ*), coefficient of determination (*R*^2^), and measurement errors were examined to evaluate the adequacy of each indicator. Standardized factor loadings of 0.50 or higher were considered acceptable, whereas values above 0.70 indicated strong indicator reliability (Hair et al., 2019).

The overall fit of the measurement model was assessed using multiple goodness-of-fit indices because no single index adequately reflects model fit. Consistent with previous methodological recommendations, a *χ*^2^/d*f* ratio ≤ 3.00, Comparative Fit Index (CFI) and Tucker–Lewis Index (TLI) values of 0.90 or higher, and Root Mean Square Error of Approximation (RMSEA) and Standardized Root Mean Square Residual (SRMR) values of 0.08 or lower were considered indicative of acceptable model fit (Brown, 2015; Hair et al., 2019; Kline, 2016).

Following the evaluation of global model fit, construct validity was assessed through convergent and discriminant validity analyses. Convergent validity was evaluated using Composite Reliability (CR) and Average Variance Extracted (AVE). CR values of 0.70 or higher and AVE values of 0.50 or higher were considered evidence of satisfactory convergent validity (Fornell and Larcker, 1981). Discriminant validity was evaluated using the Fornell–Larcker criterion, whereby the square root of the AVE for each construct should exceed its correlations with the remaining latent constructs (Fornell and Larcker, 1981; Hair et al., 2019).

After establishing the adequacy of the measurement model, the structural model was evaluated to test the hypotheses proposed in the conceptual framework. Structural relationships among latent variables were examined using standardized path coefficients (*β*), standard errors, critical ratios (t-values), and probability values (*p*-values). The explanatory power of the model was assessed using the coefficient of determination (R^2^) for each endogenous construct. According to Hair et al. (2019), higher *R*^2^ values indicate greater explanatory capability of the proposed structural model.

The hypothesized indirect association through psychological well-being was examined using a bias-corrected bootstrap procedure with 5,000 resamples. Bootstrap analysis is recommended because it does not assume normality of the indirect effect distribution and provides more accurate confidence intervals than traditional approaches such as the Sobel test (Hayes, 2018; Preacher and Hayes, 2008). The indirect effect was considered statistically significant when the 95% bootstrap confidence interval did not include zero.

All statistical analyses were conducted using IBM SPSS Statistics 22 for descriptive statistics, reliability analyses, and preliminary assumption testing. LISREL 8.80 was used to perform the confirmatory factor analysis and structural equation modeling.

## 3 Results

### 3.1 Descriptive statistics and correlations

Table 1 presents the descriptive statistics, internal consistency coefficients, and Pearson correlation coefficients for the study variables. As shown in the table, classroom teachers reported relatively high levels of job satisfaction (*M* = 3.94, SD = 0.45), psychological well-being (*M* = 4.28, SD = 0.45), and movement facilitation self-efficacy (*M* = 4.10, SD = 0.56).

**Table 1:** Descriptive statistics, reliability coefficients, and correlations among the study variables (*N* = 320).

Variable	*M*	SD	1	2	3	*α*
1. Job satisfaction	3.94	0.45	—			0.90
2. Psychological well-being	4.28	0.45	0.607**	—		0.80
3. Self-efficacy	4.10	0.56	0.528**	0.564**	—	0.94

**p < 0.01.

The internal consistency coefficients indicated satisfactory reliability for all measurement instruments. Cronbach’s alpha values ranged from 0.80 to 0.94, exceeding the recommended minimum threshold of 0.70, thereby demonstrating good to excellent internal consistency (Hair et al., 2019).

Pearson correlation analysis revealed statistically significant, positive, and moderate relationships among all study variables (*p* < 0.01). Job satisfaction was positively associated with psychological well-being (*r* = 0.607), indicating that teachers with higher levels of job satisfaction tended to report better psychological well-being. Job satisfaction was also positively correlated with movement facilitation self-efficacy (*r* = 0.528), while psychological well-being demonstrated a positive relationship with movement facilitation self-efficacy (*r* = 0.564).

None of the intercorrelations exceeded 0.80, suggesting that multicollinearity was not a concern and that the variables represented conceptually distinct constructs (Hair et al., 2019). Overall, the correlation coefficients provided preliminary support for the hypothesized relationships and justified proceeding with the structural equation modeling analyses.

### 3.2 Measurement model (CFA)

Prior to evaluating the structural relationships, the measurement model was examined using confirmatory factor analysis (CFA). Although the MSQ and TETPPACS comprise theoretically meaningful subdimensions, the measurement model was specified at the overall construct level in accordance with the primary theoretical objective of the present study. Specifically, the study focused on the structural relationships among overall job satisfaction, psychological well-being, and movement facilitation self-efficacy rather than on differential relationships among their respective subdimensions. Accordingly, the subdimensions of the MSQ and TETPPACS were acknowledged as theoretically meaningful components of their broader constructs, while the structural model was specified using the three overall constructs. This construct-level specification should not be interpreted as implying that the respective subdimensions are psychometrically equivalent or unidimensional; rather, subdimension-specific structural relationships were beyond the scope of the present study.

#### 3.2.1 Model fit indices

The fit of the construct-level measurement model comprising job satisfaction, psychological well-being, and movement facilitation self-efficacy was evaluated using CFA. The goodness-of-fit indices obtained for this specification are presented in Table 2.

**Table 2:** Goodness-of-fit indices for the construct-level measurement model.

Fit index	Model value	Recommended criterion	Interpretation	Reference
*χ*^2^ (d*f* = 964)	4049.72	—	Significant^†^	Kline (2016)
*χ*^2^/d*f*	4.20	≤5.00	Acceptable	Kline (2016)
RMSEA (90% CI)	0.06 (0.06–0.07)	≤0.08	Acceptable	Hu and Bentler (1999); Kline (2016)
CFI	0.93	≥0.90	Acceptable	Hu and Bentler (1999)
TLI (NNFI)	0.94	≥0.90	Acceptable	Hu and Bentler (1999)
NFI	0.92	≥0.90	Acceptable	Bentler and Bonett (1980)
IFI	0.93	≥0.90	Acceptable	Bollen (1989)
SRMR	0.07	≤0.08	Acceptable	Hu and Bentler (1999)
GFI	0.93	≥0.90	Acceptable	Jöreskog and Sörbom (1993)
AGFI	0.92	≥0.90	Acceptable	Jöreskog and Sörbom (1993)

*χ*^2^, Chi-square statistic; d*f*, degrees of freedom; RMSEA, Root Mean Square Error of Approximation; CFI, Comparative Fit Index; TLI, Tucker–Lewis Index; NNFI, Non-Normed Fit Index; NFI, Normed Fit Index; IFI, Incremental Fit Index; SRMR, Standardized Root Mean Square Residual; GFI, Goodness-of-Fit Index; AGFI, Adjusted Goodness-of-Fit Index. ^†^The Chi-square statistic is highly sensitive to sample size; therefore, model evaluation should be based on multiple fit indices rather than *χ*^2^ alone (Kline, 2016).

The measurement model demonstrated an acceptable overall fit to the observed data. The chi-square statistic was statistically significant (*χ*^2^ = 4049.72, d*f* = 964), which is expected given the sensitivity of the chi-square test to sample size. Therefore, additional fit indices were considered when evaluating model adequacy (Kline, 2016). The *χ*^2^/d*f* ratio was 4.20. Although this value exceeded the more stringent criterion of 3.00, the overall adequacy of the measurement model was evaluated on the basis of multiple fit indices rather than relying on the *χ*^2^/d*f* ratio alone.

Among the absolute fit indices, the RMSEA value (0.06, 90% CI [0.06, 0.07]) indicated a good approximation of model fit. Similarly, the GFI (0.93) and AGFI (0.92) values exceeded the recommended minimum criterion of 0.90, suggesting that the proposed model adequately reproduced the observed covariance matrix (Jöreskog and Sörbom, 1993).

The incremental fit indices also supported the adequacy of the measurement model. The CFI (0.93), TLI (0.94), NFI (0.92), and IFI (0.93) values all exceeded the recommended threshold of 0.90, indicating acceptable model fit according to established recommendations (Bentler and Bonett, 1980; Bollen, 1989; Hu and Bentler, 1999).

The SRMR value (0.07) was also within the recommended threshold, further supporting the adequacy of the measurement model. Considering the acceptable values obtained for RMSEA, CFI, TLI, GFI, and AGFI, the overall pattern of fit indices indicates that the measurement model provides an acceptable representation of the relationships among the latent constructs and was therefore considered appropriate for subsequent structural model evaluation (Hair et al., 2019; Kline, 2016).

#### 3.2.2 Standardized factor loadings

Within the construct-level measurement specification adopted for the present study, standardized factor loadings were examined to evaluate the extent to which the observed indicators represented the three broader constructs included in the structural model. As presented in Table 3, all retained indicators loaded significantly on their specified overall constructs (*p* < 0.001). The standardized factor loadings ranged from 0.54 to 0.88, exceeding the recommended minimum threshold of 0.50 and indicating generally adequate indicator–construct relationships within the specified model (Hair et al., 2019).

**Table 3:** Standardized indicator loadings for the construct-level measurement specification.

Construct	Number of items	*λ*Min	*λ*Max
Job satisfaction	20	0.56	0.84
Psychological well-being	8	0.54	0.81
Self-efficacy	18	0.61	0.88

All standardized factor loadings were statistically significant (*p* < 0.001).

All indicators demonstrated statistically significant and practically meaningful relationships with their specified overall constructs, providing support for indicator reliability within the construct-level measurement model. No standardized loading fell below the adopted 0.50 criterion; therefore, no items were removed on the basis of factor loadings.

These findings should be interpreted within the construct-level analytical framework adopted for the present study. The measurement specification was not intended to test whether the established subdimensions of the MSQ and TETPPACS could be replaced by strictly unidimensional structures. Rather, it was used to represent the broader constructs of job satisfaction, psychological well-being, and movement facilitation self-efficacy in accordance with the construct-level hypotheses of the study. Accordingly, the results support the adequacy of the indicator–construct relationships within the specified model but should not be interpreted as evidence against the established subdimensional structures of the MSQ and TETPPACS.

#### 3.2.3 Reliability and construct validity

The reliability and construct validity results of the measurement model are presented in Table 4. Internal consistency was evaluated using Composite Reliability (CR), whereas convergent validity was assessed using the Average Variance Extracted (AVE). Discriminant validity was examined using the Fornell–Larcker criterion (Fornell and Larcker, 1981).

**Table 4:** Reliability, convergent validity, and discriminant validity of the measurement model.

Variable	CR	AVE	JT	PWB	TETPPAC
JT	0.91	0.52	**0.72**		
PWB	0.86	0.50	0.61	**0.71**	
TETPPAC	0.95	0.55	0.53	0.56	**0.74**

Path/Effect		*β*	*t*	*p*	95% CI	*R* ^2^
JT → PWB	Direct	0.64	10.46	<0.001	—	0.40
PWB → TETPPAC	Direct	0.39	5.14	<0.001	—	
JT → TETPPAC	Direct	0.27	3.88	<0.001	—	
JT → PWB → TETPPAC	Indirect	0.25	—	<0.001	[0.17, 0.34]	
JT → TETPPAC	Total	0.52	—	<0.001	—	0.39

CR, composite reliability; AVE, average variance extracted. Bold diagonal values represent the square roots of the AVE and were used to assess discriminant validity according to the Fornell–Larcker criterion (Fornell and Larcker, 1981).

As presented in Table 4, the CR values ranged from 0.86 to 0.95, exceeding the recommended minimum threshold of 0.70 and indicating satisfactory internal consistency for all latent constructs. Likewise, the AVE values ranged from 0.50 to 0.55, meeting or exceeding the recommended criterion of 0.50. These findings support the convergent validity of the measurement model (Fornell and Larcker, 1981; Hair et al., 2019).

Discriminant validity was evaluated using the Fornell–Larcker criterion, whereby the square root of the AVE for each construct should exceed its correlations with the remaining constructs. As shown in Table 4, the square roots of the AVE values were 0.72 for Job Satisfaction, 0.71 for Psychological Well-Being, and 0.74 for Movement facilitation self-efficacy. In each case, these values were greater than the corresponding inter-construct correlations, demonstrating satisfactory discriminant validity.

Overall, the reliability and validity findings indicate that the measurement model possesses adequate psychometric properties and provides a sound basis for testing the structural relationships proposed in the study.

### 3.3 Structural model

Following confirmation of the adequacy of the measurement model, the hypothesized structural model was evaluated. The standardized path coefficients, significance levels, explained variance (*R*^2^), and bootstrap results for the indirect association are presented in Table 5.

**Table 5:** Structural model results.

Path	*β*	*t*	*p*	95% CI	*R* ^2^
JT → PWB	0.64	10.46	<0.001	—	0.40
PWB → TETPPAC	0.39	5.14	<0.001	—	
JT → TETPPAC	0.27	3.88	<0.001	—	
JT → PWB → TETPPAC (indirect)	0.25	—	<0.001	[0.17, 0.34]	
JT → TETPPAC (total effect)	0.52	—	<0.001	—	0.39

Indirect effects were estimated using the bias-corrected bootstrap procedure with 5,000 resamples.

As hypothesized, job satisfaction was significantly and positively associated with psychological well-being (*β* = 0.64, *t* = 10.46, *p* < 0.001), with the model explaining 40% of the variance in psychological well-being (*R*^2^ = 0.40). This result indicates that teachers reporting higher levels of job satisfaction also tended to report higher levels of psychological well-being.

Psychological well-being was significantly and positively associated with movement facilitation self-efficacy (*β* = 0.39, *t* = 5.14, *p* < 0.001). In addition, job satisfaction was significantly and positively associated with movement facilitation self-efficacy (*β* = 0.27, *t* = 3.88, *p* < 0.001), indicating that teachers reporting higher levels of job satisfaction also tended to report greater confidence in facilitating students’ participation in physical activity during physical education and games lessons.

The indirect association between job satisfaction and movement facilitation self-efficacy through psychological well-being was examined using a bias-corrected bootstrap procedure with 5,000 resamples. The indirect effect was statistically significant (*β* = 0.25, 95% CI [0.17, 0.34]). Because the confidence interval did not include zero, the indirect association was considered statistically significant (Preacher and Hayes, 2008).

The total association between job satisfaction and movement facilitation self-efficacy was *β* = 0.52 (*p* < 0.001), and the proposed structural model explained 39% of the variance in movement facilitation self-efficacy (*R*^2^ = 0.39). Collectively, these findings indicate that job satisfaction was associated with movement facilitation self-efficacy both directly and indirectly through psychological well-being.

#### 3.3.1 Hypothesis testing

To facilitate the interpretation of the structural model results, the outcomes of the hypothesis testing are summarized in Table 6. Each hypothesis was evaluated based on the significance and direction of the standardized path coefficients obtained from the structural equation modeling analysis.

**Table 6:** Hypothesis testing results.

Hypothesis	Proposed relationship	Decision
H1	Job satisfaction → Psychological well-being	Supported
H2	Psychological well-being → Movement facilitation self-efficacy	Supported
H3	Job satisfaction → Movement facilitation self-efficacy	Supported
H4	Job satisfaction → Psychological well-being → Movement facilitation self-efficacy	Supported (significant indirect association)

All four hypotheses proposed in the conceptual model were supported. Job satisfaction was positively associated with psychological well-being (H1) and movement facilitation self-efficacy (H3), while psychological well-being was positively associated with movement facilitation self-efficacy (H2). Furthermore, a statistically significant indirect association between job satisfaction and movement facilitation self-efficacy through psychological well-being was observed (H4). Because both the direct association and the indirect association were statistically significant, the findings indicate that psychological well-being statistically accounted for part of the association between job satisfaction and movement facilitation self-efficacy (Zhao et al., 2010).

The structural model presented in Figure 1 illustrates the direct and indirect associations among job satisfaction, psychological well-being, and movement facilitation self-efficacy. Job satisfaction was strongly and positively associated with psychological well-being (*β* = 0.64) and was significantly associated with movement facilitation self-efficacy (*β* = 0.27). Psychological well-being was also positively associated with movement facilitation self-efficacy (*β* = 0.39). Bootstrap analysis further indicated a statistically significant indirect association between job satisfaction and movement facilitation self-efficacy through psychological well-being (*β* = 0.25, 95% CI [0.17, 0.34]), supporting the hypothesized indirect association. Overall, the structural model accounted for 40% of the variance in psychological well-being and 39% of the variance in movement facilitation self-efficacy.

**Figure 1:**
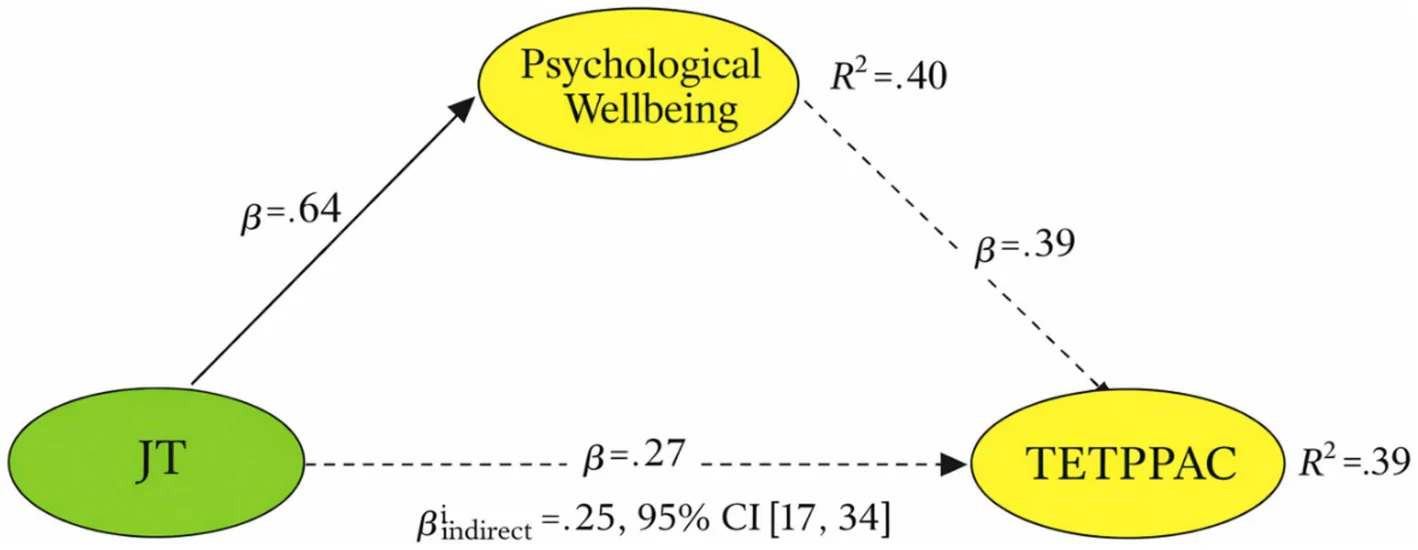
Structural equation model of the relationships among job satisfaction, psychological well-being, and movement facilitation self-efficacy.

## 4 Discussion

This study examined the relationships among classroom teachers’ job satisfaction, psychological well-being, and movement facilitation self-efficacy in physical education and games lessons within the framework of a structural equation model. The findings revealed that the model showed acceptable fit at both the measurement and structural levels and confirmed the theoretically expected relationships between the variables.

First, job satisfaction was significantly and positively associated with psychological well-being (*β* = 0.64), indicating that teachers reporting higher levels of job satisfaction also tended to report better psychological functioning. This finding is consistent with meta-analytic and theoretical studies reporting a strong relationship between job satisfaction and subjective well-being (Judge et al., 2001; Diener et al., 2018). In the educational context, previous research has also linked teachers’ job satisfaction with greater psychological resilience and lower levels of burnout (Skaalvik and Skaalvik, 2017). Within this framework, the current findings suggest that teachers’ job satisfaction is closely associated not only with organizational outcomes but also with their psychological resilience and functioning.

Second, psychological well-being was significantly and positively associated with movement facilitation self-efficacy (*β* = 0.39), a finding that is consistent with Bandura’s (1997) Social Cognitive Theory. According to Bandura, self-efficacy beliefs are related not only to an individual’s knowledge and skills but also to emotional and physiological states. Individuals with more positive psychological and emotional states may perceive themselves as more capable of performing challenging tasks. The literature on teacher self-efficacy similarly reports meaningful associations between psychological well-being and self-efficacy (Zee and Koomen, 2016). The present findings extend this association to the context of physical education and games lessons, specifically to teachers’ confidence in facilitating students’ participation in physical activity.

Third, the findings indicated both a significant direct association between job satisfaction and movement facilitation self-efficacy (*β* = 0.27) and a significant indirect association through psychological well-being (*β* = 0.25). The significant indirect association indicates that psychological well-being statistically accounts for part of the association between job satisfaction and movement facilitation self-efficacy. However, because the direct association remained statistically significant, the relationship between job satisfaction and movement facilitation self-efficacy was not accounted for solely by psychological well-being. Statistically, this pattern is consistent with the complementary mediation framework described by Zhao et al. (2010); however, given the cross-sectional design of the present study, this pattern should be interpreted as evidence of direct and indirect associations rather than as a causal mediating mechanism.

Regarding the explanatory power of the model, the model accounted for 40% of the variance in psychological well-being and 39% of the variance in movement facilitation self-efficacy. According to Hair et al. (2019), these values indicate a moderately strong level of explanatory power in the context of social sciences. Taken together, these findings suggest that movement facilitation self-efficacy is meaningfully associated not only with teachers’ professional experiences but also with their psychosocial resources.

The results obtained reveal that school-based physical activity policies should not be limited to curriculum and program arrangements. Supporting teachers’ job satisfaction and psychological well-being may provide favorable psychosocial conditions for pedagogical practices that encourage physical activity. In this context, teacher well-being can be considered a dimension of physical activity policies that should not be overlooked.

Furthermore, the findings suggest that teachers’ professional well-being can serve as a psychological resource that enables behaviorally intensive pedagogical practices, especially in movement-based teaching environments.

In conclusion, this study contributes to the literature by testing the relationship between teachers’ psychological well-being and movement facilitation self-efficacy within a comprehensive structural model. The findings provide empirical evidence that job satisfaction and psychological well-being are important psychosocial correlates of teachers’ confidence in implementing practices that encourage physical activity.

### 4.1 Limitations and suggestions for future research

Despite the theoretical and practical contributions of the present study, several limitations should be considered when interpreting the findings.

First, this study employed a cross-sectional research design, which limits the ability to draw causal conclusions regarding the relationships among job satisfaction, psychological well-being, and movement facilitation self-efficacy. Although the proposed structural model was theoretically grounded and demonstrated satisfactory empirical fit, longitudinal or experimental studies are needed to examine the temporal and causal nature of these relationships more rigorously (Maxwell and Cole, 2007).

Second, all data were collected using self-report questionnaires, which may increase the possibility of response bias, including social desirability and common method variance. Although procedural precautions were implemented during data collection and the Harman single-factor test suggested that common method bias was not a serious concern, future studies could strengthen methodological rigor by incorporating multiple data sources, such as classroom observations, peer evaluations, school administrator assessments, or objective indicators of classroom physical activity.

Third, the study was conducted exclusively with classroom teachers working in public primary schools in Ankara, Türkiye. Consequently, caution should be exercised when generalizing the findings to private schools, different educational levels, rural settings, or other cultural contexts. Replication studies involving more diverse samples would improve the external validity and generalizability of the proposed model.

Fourth, the conceptual model included only three psychosocial constructs. Although the model explained a substantial proportion of the variance in both psychological well-being and movement facilitation self-efficacy, additional organizational and contextual variables may further enhance explanatory power. Future studies may consider examining factors such as school leadership, organizational support, school climate, teacher autonomy, work engagement, occupational stress, burnout, and organizational commitment within more comprehensive structural models.

Building upon these limitations, future research may employ longitudinal, multilevel, or mixed-methods research designs to obtain a more comprehensive understanding of the mechanisms linking teachers’ occupational well-being and movement-oriented instructional practices. Furthermore, intervention studies evaluating whether programs designed to improve teachers’ job satisfaction and psychological well-being lead to increased movement facilitation self-efficacy would provide stronger evidence regarding the practical implications of the proposed theoretical model. Future research may also examine additional organizational and contextual variables, including school leadership, organizational support, school climate, teacher autonomy, work engagement, burnout, and organizational commitment, all of which have previously been identified as important determinants of teacher well-being and instructional effectiveness (Collie, 2021; Skaalvik and Skaalvik, 2017; Zee and Koomen, 2016). Such investigations would contribute to a more comprehensive understanding of the organizational and psychological conditions that support movement-oriented teaching and may further improve the explanatory power of future theoretical models.

These findings should also be interpreted within the specific context of primary education in Türkiye. The participants in this study were classroom teachers working in public primary schools in Ankara who were responsible for delivering physical education and games lessons. In this context, movement-oriented instruction constitutes one component of classroom teachers’ broader instructional responsibilities rather than a separate specialist teaching role. Therefore, their confidence in facilitating physical activity may be closely associated not only with pedagogical competence but also with the occupational and psychological conditions under which they perform their teaching responsibilities. The significant relationships observed among job satisfaction, psychological well-being, and movement facilitation self-efficacy suggest that efforts to strengthen physical activity practices in Turkish primary schools may benefit from considering teacher well-being alongside curriculum implementation and instructional support. In particular, school-level practices that support teachers’ job satisfaction and psychological well-being may provide a more favorable professional context for the effective implementation of physical education and games lessons.

Finally, although the present study focused on overall job satisfaction and movement facilitation self-efficacy in accordance with its construct-level theoretical framework, both instruments include theoretically meaningful subdimensions. Future studies should examine whether the observed direct and indirect associations differ across intrinsic and extrinsic job satisfaction and across the student, institutional, and educational dimensions of movement facilitation self-efficacy.

### 4.2 Application and policy recommendations

The findings of this study have important implications for educational practice, school leadership, and policy development. Since job satisfaction and psychological well-being were found to be significantly associated with teachers’ movement facilitation self-efficacy, interventions aimed at supporting movement-oriented teaching may benefit from considering teachers’ job satisfaction and psychological well-being alongside curriculum design and instructional resources.

First, school administrators should establish organizational practices that enhance teachers’ job satisfaction by promoting supportive leadership, professional recognition, participatory decision-making, and positive working conditions. Previous research has shown that supportive school environments contribute not only to teachers’ well-being but also to their instructional effectiveness and long-term professional commitment (Collie, 2021). Strengthening these organizational conditions may therefore indirectly enhance teachers’ confidence in implementing movement-based instructional practices.

Second, teacher professional development programs should integrate psychological well-being with pedagogical competence. In-service training should include not only movement-based teaching strategies but also topics such as stress management, emotional resilience, work engagement, and positive occupational functioning. Supporting teachers’ psychological resources may increase their willingness and confidence to implement physically active learning environments, particularly when they encounter institutional or instructional barriers.

Third, educational policymakers should recognize teacher well-being as an essential component of comprehensive school physical activity initiatives. National and local policies frequently emphasize increasing students’ physical activity opportunities; however, relatively limited attention has been devoted to the psychosocial conditions that enable teachers to implement these initiatives effectively. The present findings suggest that investments in teacher well-being may indirectly strengthen the implementation and sustainability of school-based physical activity programs.

Fourth, teacher education institutions may consider incorporating movement-oriented pedagogy and physical activity promotion into pre-service teacher education curricula. Equipping future classroom teachers with both instructional competencies and psychological coping resources before entering the profession may strengthen their preparedness to integrate physical activity into everyday classroom practice.

Finally, collaboration among school leaders, educational authorities, and public health organizations may facilitate the development of multidisciplinary programs aimed at improving both teacher well-being and student physical activity participation. Such collaborative initiatives may contribute to healthier school environments by simultaneously supporting teacher development and promoting active learning experiences for students.

Overall, the present findings indicate that strengthening teachers’ occupational and psychological resources should be considered a strategic investment not only for improving teacher well-being but also for enhancing the successful implementation of movement-oriented educational practices in primary schools.

## 5 Conclusion

This study examined the relationships among classroom teachers’ job satisfaction, psychological well-being, and movement facilitation self-efficacy within the context of physical education and games lessons. Using a structural equation modeling approach, the findings demonstrated that job satisfaction was positively associated with movement facilitation self-efficacy both directly and indirectly through psychological well-being. These results indicate that psychological well-being statistically accounted for part of the association between teachers’ job satisfaction and movement facilitation self-efficacy.

The study contributes to the literature by extending research on teacher self-efficacy beyond general instructional contexts and focusing specifically on movement facilitation self-efficacy among primary education classroom teachers. The findings suggest that teachers’ confidence in promoting physical activity is associated not only with pedagogical competence but also with job satisfaction and psychological functioning. In doing so, the study integrates perspectives from organizational psychology, teacher well-being, and school-based physical activity research within a single explanatory framework.

From a practical perspective, the findings suggest that supporting teachers’ occupational well-being may be associated with more favorable conditions for creating physically active learning environments. Accordingly, initiatives aimed at improving school-based physical activity practices may benefit from considering teachers’ job satisfaction and psychological well-being alongside instructional and organizational factors.

Overall, the study demonstrates that teachers’ occupational and psychological resources are meaningfully associated with movement facilitation self-efficacy. By providing empirical evidence for the direct and indirect pathways linking job satisfaction, psychological well-being, and self-efficacy, the study offers a foundation for future research and interventions designed to strengthen both teacher well-being and movement-oriented teaching practices in primary education.

## Data Availability

The raw data supporting the conclusions of this article will be made available by the authors, without undue reservation.
